# Renal ^123^I-MIBG Uptake before and after Live-Donor Kidney Transplantation

**DOI:** 10.3390/diagnostics10100802

**Published:** 2020-10-09

**Authors:** Thomas Rasmussen, Robin de Nijs, Lene Kjær Olsen, Anne-Lise Kamper, Lia Evi Bang, Marie Frimodt-Møller, Henning Kelbæk, Søren Schwartz Sørensen, Andreas Kjær, Bo Feldt-Rasmussen, Jesper Hastrup Svendsen, Philip Hasbak

**Affiliations:** 1 Departments of Clinical Physiology, Nuclear Medicine & PET and Cluster for Molecular Imaging, Copenhagen University Hospital, Rigshospitalet, 2100 Copenhagen, Denmark; robin.de.nijs@regionh.dk (R.d.N.); andreas.kjaer@regionh.dk (A.K.); philip.hasbak@regionh.dk (P.H.); 2 Department of Nephrology, Copenhagen University Hospital, Rigshospitalet, 2100 Copenhagen, Denmark; lenekjaerolsen@gmail.com (L.K.O.); anne-lise.kamper@regionh.dk (A.-L.K.); soeren.schwartz.soerensen@regionh.dk (S.S.S.); bo.feldt-rasmussen@regionh.dk (B.F.-R.); 3 Department of Cardiology, Copenhagen University Hospital, Rigshospitalet, 2100 Copenhagen, Denmark; lia.evi.bang@regionh.dk (L.E.B.); jesper.hastrup.svendsen@regionh.dk (J.H.S.); 4 Steno Diabetes Center, Gentofte University Hospital, 2820 Gentofte, Denmark; marie.frimodt-moeller@regionh.dk; 5 Department of Cardiology, Zealand University Hospital, 4000 Roskilde, Denmark; h.kelbaek@dadlnet.dk; 6 Department of Clinical Medicine, Faculty of Health and Medical sciences, University of Copenhagen, 2100 Copenhagen, Denmark

**Keywords:** renal nervous activity, ^123^I-MIBG scintigraphy, renal transplantation, sympathetic nervous system, live kidney donor

## Abstract

Increased sympathetic activity is suggested to be part of the pathogenesis in several diseases. Methods to evaluate sympathetic activity and renal nervous denervation procedural success are lacking. Scintigraphy using the norepinephrine analog Iodine-123 Metaiodobenzylguanidine (^123^I-MIBG) might provide information on renal sympathetic nervous activity. Renal transplantation induces complete denervation of the kidney and as such represents an ideal model to evaluate the renal ^123^I-MIBG scintigraphy method. The aim of this study was to evaluate whether renal ^123^I-MIBG scintigraphy can detect changes in renal sympathetic nervous activity following renal transplantation. Renal ^123^I-MIBG scintigraphy was performed in eleven renal transplant recipients at 1, 3, and 6 months following transplantation and in their respective living donors prior to their kidney donation. Relative uptake as well as washout was obtained. In transplanted patients, the relative 4 h uptake of ^123^I-MIBG, as measured by the kidney/background ratio, was 2.7 (0.4) (mean (SD)), 2.7 (0.5), and 2.5 (0.4) at 1, 3, and 6 months post-transplantation, respectively, as compared with the 4.0 (0.4) value in the donor kidney before donor nephrectomy (*p* < 0.01). There was no significant change in washout-rate between pre-transplantation and any of the follow-up time points. Living donor kidney transplantation was at 6 months post transplantation, associated with an almost 40% reduction in the relative 4 h ^123^I-MIBG uptake of the kidney. Further studies will help to fully establish its implications as a marker of renal innervation or denervation.

## 1. Introduction

The activity of sympathetic renal nerves plays an important role in chronic kidney disease [1,2,3], renal transplantation [4,5,6], and in hypertension when hypertension is treated by renal denervation [7,8,9]. Stimulation of renal efferent sympathetic nerves increases sodium retention and renin release and reduces renal blood flow [10,11].

**Figure 1:**
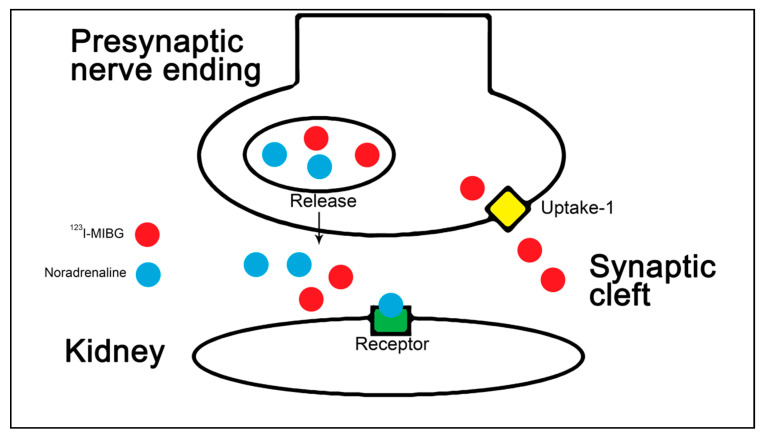
Iodine-123 Metaiodobenzylguanidine (^123^I-MIBG) uptake and release. The figure illustrates how ^123^I-MIBG in the presynaptic nerve terminal is taken up via the uptake-1 transport mechanism, stored as granules within vesicles, and then released. The scintigraphic measurement over the kidney at different time points provides information regarding the early uptake and the functionality.

Iodine-123 Metaiodobenzylguanidine (^123^I-MIBG) is an analog to norepinephrine and is taken up by the uptake-1 transport mechanism, also known as the norepinephrine transporter, in the presynaptic nerve terminal, and it is stored in the neurosecretory granules, resulting in a higher concentration compared with that in cells of tissues with lower sympathetic activity [12,13,14] (Figure 1). ^123^I-MIBG is predominantly excreted as an unchanged compound by the kidneys, with a rapid initial clearance followed by an almost constant plasma level between 1 and 24 h after injection of ^123^I-MIBG [15,16,17]. ^123^I-MIBG is a substrate of renal organic cation transporters, suggesting that it undergoes tubular secretion [18]. Therefore, renal ^123^I-MIBG is composed of four components: vascular, urinary, parenchymal, and neuronal. The radioactive labeling of ^123^I-MIBG enables scintigraphic imaging of presynaptic anatomy and function. ^123^I-MIBG is used clinically for diagnosis and localization of pheochromocytoma [19] and has been proposed as a prognostic marker in chronic heart failure [20].

^123^I-MIBG uptake supposedly visualizes reinnervation over time in heart transplant patients [21], and renal tracer activity has been demonstrated in patients with severe renal artery stenosis or renal artery compression [22,23]. A study of renal ^123^I-MIBG scintigraphy performed in 12 kidney transplant recipients has shown an association between time from transplantation and renal ^123^I-MIBG uptake parameters, suggesting functional re-innervation [24]. In a case-study of a fibromuscular dysplasia-induced renal artery stenosis, the relative ^123^I-MIBG uptake in the auto transplanted kidney was reduced by 30% at 2 weeks after the procedure [25]. In another study of renal ^123^I-MIBG scintigraphy performed before and six weeks after renal denervation for treatment of treatment-refractory essential hypertension, there was no significant change in renal ^123^I-MIBG relative uptake or washout [26]. Neuroanatomically, following the nerve transection that occurs in kidney transplantation, the distal part of the detached axon undergoes a Wallerian degeneration, which is a rapid, within days, fragmentation and cytoskeletal breakdown that occurs along the full length of the axon; however, overall, the periaxonal structure is maintained [27,28]. It is known that re-innervation of transplanted kidneys occurs, and partial reinnervation has been confirmed in histological studies [5,6]. In human renal allografts, surgically removed or post-mortem, histological evidence of axon regeneration was evident already 8 months after transplantation [6], whereas the degree of functionality of this regeneration of axons is questionable [4].

The aim of this study was to evaluate whether renal ^123^I-MIBG could detect any changes in renal sympathetic nervous activity following renal transplantation from living donors. Our hypothesis was that the denervated transplanted kidney would display a markedly reduced ^123^I-MIBG uptake compared to that found pre-transplantation.

## 2. Materials and Methods

### 2.1. Subjects

We prospectively included renal transplantation recipients (*n* = 11) and their respective live donors prior to kidney transplantation at Copenhagen University Hospital, Rigshospitalet, Denmark, from April 2014 to May 2015. Whereas 41 live kidney transplantations were performed in that period, limitation of the capacity to perform ^123^I-MIBG scintigraphy restricted us to invite only 18 donor/recipient duos to participate, whereof 5 declined the invitation and 2 had their transplantation canceled. It was a clinical decision not to pause medication that could potentially influence ^123^I-MIGB uptake such as beta-blockers, calcium channel blockers, etc. Inclusion criteria were age ≥18 years, eligibility for kidney donation/transplantation (donor/recipient in pairs) and clinically stable condition. Reason for withdrawal during the study was suspicion of or confirmation of acute rejection of the transplanted kidney at the follow-up time points.

### 2.2. Ethics

The study was approved by the Scientific-Ethics Committee for the Capital Region of Denmark (H-3-2013-190) and the Danish Data Protection Agency (30-1311), approved on 15th of January 2014. All participants signed informed consent forms prior to inclusion.

### 2.3. Image Acquisition

Renal ^123^I-MIBG scintigraphy was performed in donors prior to transplantation and in their respective recipients 1, 3, and 6 months after renal transplantation. Thyroid iodine uptake was blocked before injection of ^123^I-MIBG by oral administration of potassium perchlorate (150 mg). Rest planar and SPECT/CT imaging were performed sequentially beginning approximately 15 min post-injection of 300–350 MBq ^123^I-MIBG (AdreView^TM^, GE Healthcare, Eindhoven, The Netherlands) followed by planar imaging at 1, 2, and 4 h post-injection. Due to the practical clinical workflow, subjects were scanned either in a dual head 16-slice Precedence single-photon emission computed tomography (SPECT)/multi-detector CT (MDCT), (Philips Medical Systems, Best, The Netherlands) or a dual head Symbia T16 SPECT/CT scanner (Siemens, Erlangen, Germany) with a 9.5 mm thick scintillation crystal. In the following sections, Philips and Siemens parameters will be stated, and if different, they will be separated by a slash. ^123^I-MIBG was imaged with a standard Anger camera using a medium-energy general purpose collimator/low penetration, MEGP/MELP, to reduce the contribution of high-energy ^123^I photon emissions, especially those at 529 keV to penetrate collimator septa [29,30]. The energy window was set at 159 keV with a width of 20%/15%. The acquisition protocol on the scanner consisted of a ten minutes planar acquisition (arms down) with matrix size 256 × 256 and 2.332/2.398 mm pixel size. Later planar acquisitions were acquired with the same settings, but with slightly different patient position. SPECT data was acquired in a 128 × 128 matrix with a pixel size of 4.664/4.796 mm at 128 angles (20 s each) in step-and-shoot mode. A low dose CT scan of the same region was acquired with 5 mm thick slices in a 512 × 512 matrix with a pixel size of 1.172/0.977 mm at 140/130 kV.

### 2.4. Image Processing and Interpretation

**Figure 2:**
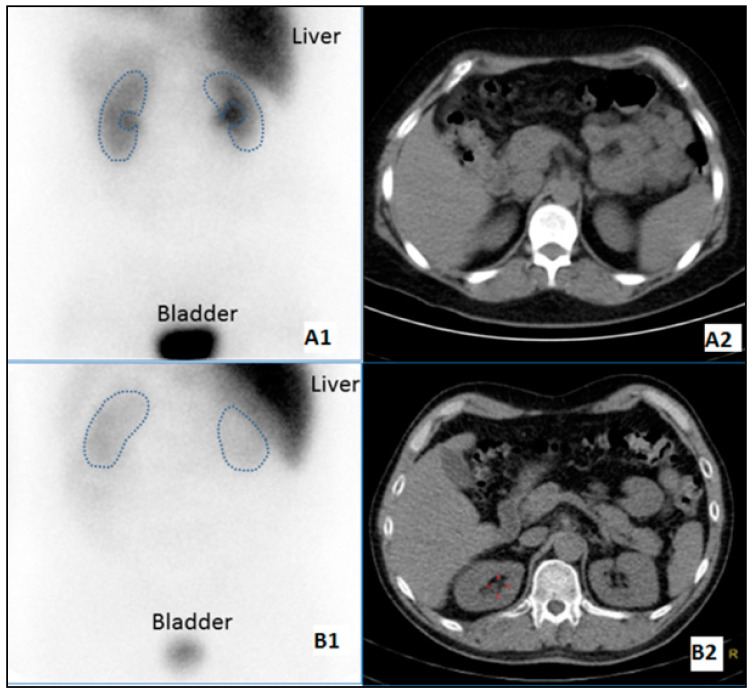
Scintigraphic visualization of ^123^I-MIBG uptake. The figure illustrates the challenge when using 2D-planar recording for drawing of regions of interest (ROIs). In (**A1**,**A2**), the right kidney is anatomically free of the liver, while the right kidney is covered by the liver in (**B1**). By using SPECT/CT for ROI drawing in (**A1**,**B2**), this overlap could be avoided (Red crosshair in (**B2**) marks the right renal pelvis). The presence of renal pelvic activity in (**A1**) (although excluded on the ROIs) makes it evident that substantial counts are due to excretory activity.

The washout rate not only assesses the ability of the kidney to retain the tracer but may also reflect competition for the uptake-1 transport mechanism by circulating norepinephrine. The early kidney/background ratio reflects receptor density and likely portrays the integrity of presynaptic nerve terminals and uptake-1 function. ^123^I-MIBG SPECT data was reconstructed using Astonish/Flash3D, Philips Healthcare, Best, The Netherlands. Regions of interest (ROIs) were drawn over the kidney(s) and background on planar images, see Figure 2.

Since the anatomical position of the kidney was different within the donor and the recipient, attenuation correction needed to be applied to enable a correct comparison of the exact same anatomical structure. An experienced nuclear physician processed all images for this study. Renal ^123^I-MIBG uptake was calculated by using mean counts per pixel obtained by manually drawn ROIs. The gluteal muscles served as reference tissue. ^123^I-MIBG image interpretation consists of quantitation of global kidney tracer uptake in reference to gluteal muscle ratio, kidney/background ratio, and retention of tracer between early and late images, that is, washout rate and regional uptake on SPECT images. The relative ^123^I-MIBG uptake was quantified by calculating the kidney/background ratio at 15 min (early), 1, 2, and 4 h (late) post tracer injection:

(1) $$\frac{\mathrm{kidney}}{\mathrm{background}}\mathrm{ratio}=\;\frac{\mathrm{kidney}\;\left(\mathrm{specific}\right)}{\mathrm{muscle}\;\left(\mathrm{non}-\mathrm{specific}\right)}$$

The washout rate between two time points following the ^123^I-MIBG infusion can be calculated by:

(2) $$\begin{array}{c} \mathrm{Washout}\;\mathrm{rate}\;\left(\%\;\mathrm{per}\;\mathrm{hour}\right)=\left(\frac{\ln\left(\frac{{\mathrm{kidney}\;\mathrm{ROI}\;\mathrm{count}}_{t=1}}{{\mathrm{kidney}\;\mathrm{ROI}\;\mathrm{count}}_{t=2}}\right)}{t2-t1}-\frac{\ln\left(2\right)}{T_{½}}\right)*100\%\\ T_{½}\;\mathrm{for}{\;}^{123}I\text{-}\mathrm{MIBG}\;=\;13.2\;h \end{array}$$

#### 2.4.1. Attenuation Correction

Attenuation correction (AC) was applied by utilizing the CT scan obtained together with the SPECT acquisition of approximately the same region of the patient. Further details of the AC can be found in Appendix A.

#### 2.4.2. Alignment

The patient remained in the same position during acquisition of the first planar image and the SPECT-CT acquisition. This allowed positioning parameters in the Digital Imaging and Communications in Medicine (DICOM) header to align the CT with the first planar image for the Philips images. Positioning information could not be stored on the Siemens scanner; therefore, the CT and planar images were aligned from the assumption that CT and planar images had identical image centers. The CT-based images (*T*_water_ and AC) were interpolated to match the pixel size of the planar images. Subsequent planar images were acquired with alignment to the first planar image by manually choosing three characteristic landmarks such as edges and notches on the anterior, posterior, and geometric mean image on both the first image and the image to be aligned. The precision of this procedure was indicated by the sample standard deviation of the mean transformation, which was less than 3 mm for every alignment.

### 2.5. Statistics

Variables were expressed as percentages or by their mean values (±SD). The hypothesis was that there would be a significant change in relative 4 h renal ^123^I-MIBG uptake from baseline to 6 months after transplantation. This change was assessed by the Student’s *t*-test for paired samples. Comparisons of kidney/background ratios at follow-up time points were assessed by one-sided ANOVA analysis. The *p* values ≤0.05 were considered significant. Statistical analysis was performed using SAS enterprise Guide 7.1.

## 3. Results

### 3.1. Demographics and Clinical Characteristics

Eleven recipients (6 men, 5 women) and their donors (7 men, 4 women) with an average age of 47 years (±16 years) and 52 years (±13 years), respectively, were included in the study (Table 1). Besides the baseline data available in Table 1, there was no history of ischemic heart disease or cerebral vascular insult in either group. One follow-up scan was missed due to technical problems. No cases of graft rejection interfered with follow-up. Further detailed information of the 11 sets of donor/recipients with concern to kidney function, medication, and co-morbidity can be found in the Supplementary Material (Table S1).

### 3.2. Studies in Donors before Nephrectomy and in Transplant Recipients

**Figure 3:**
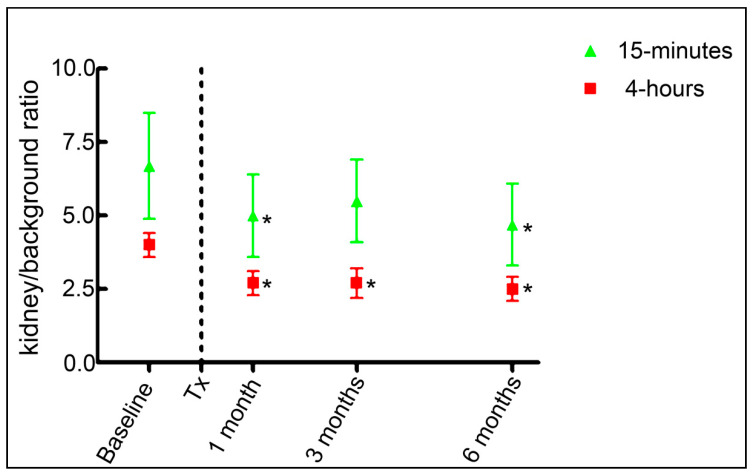
Relative ^123^I-MIBG uptake over time. Results are given as mean and standard deviation. Baseline indicates the value of the kidney within the donor, and follow-up time points are the same kidney within the recipient. 15-minutes and 4-hours indicates the relative ^123^I-MIBG uptake at these time points following intravenous injection of ^123^I-MIBG. * indicates that the change is statistically significant (*p* < 0.05) from the baseline value. Tx; kidney transplantation.

Within the group of donors, the relative renal ^123^I-MIBG uptake at 15 min was 6.8 and at 4 h 4.0. The relative renal ^123^I-MIBG uptake, kidney/background ratio, for the donor kidney at baseline, 1, 3, and 6 months at both 15 min and 4 h is visualized in Figure 3 and all results from 15 min to 4 h are listed in Table 2.

**Figure 4:**
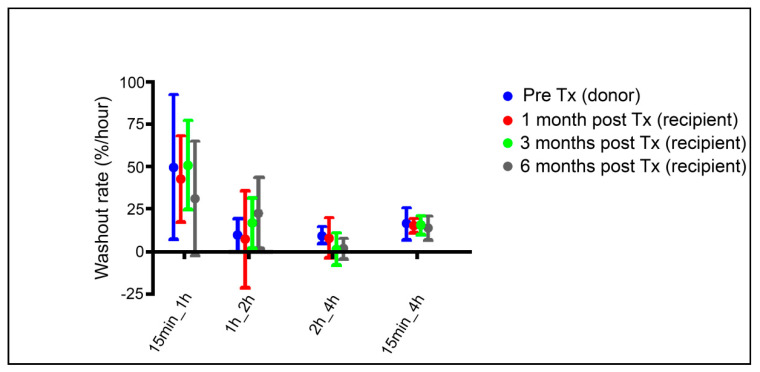
Washout rate of ^123^I-MIBG over time. Results are given as mean and standard deviation. There was no statistically significant change within any of the respective time intervals between baseline and times of follow up. Tx; kidney transplantation. The large difference between the 15 min to 1 h results compared to the other time intervals indicates that these early results reflect predominantly renal clearance of Metaiodobenzylguanidine (MIBG) via glomerular filtration rather than washout from sympathetic neurons.

The kidney/background ratio at 15 min post ^123^I-MIBG injection at pre-transplantation compared to that of the follow-up time points was significantly reduced at 1 and 6 months post-transplantation (*p* < 0.05). The 4 h kidney/background ratio was significantly decreased at 1, 3, and 6 (*p* < 0.005) months after transplantation compared to that pre-transplantation within the donor. Our results show a 29% to 33% reduction accordingly at 1 and 3 months, and a 41% reduction at 6 months in renal ^123^I-MIBG 4 h relative uptake following transplantation. Kidney/background ^123^I-MIBG 4 h uptake at 1, 3, and 6 months did not differ significantly from each other (*p* = 0.5). Washout rate was calculated for 5 individual time intervals. There were no statistical differences in ^123^I-MIBG washout rates (15 min to 1 h; 15 min to 2 h; 15 min to 4 h; 1 to 2 h; or 2 to 4 h between pre-transplantation and 1, 3, and 6 months postoperatively) see Table 2 and Figure 4 for details.

## 4. Discussion

We hypothesized that the denervated transplanted kidney would display a markedly lower ^123^I-MIBG uptake compared to pre-transplantation due to the presumed lack of axonal neuronal input. Indeed, we found that the transplanted kidney/background ratio was reduced by 30–40% compared to the innervated kidney. These findings are intriguing; however, we cannot differentiate between the vascular, urinary, parenchymal, and neuronal components of the renal ^123^I-MIBG uptake. The compensatory hyperfiltration of the transplanted kidney might be a contributing component that would have to be considered in future studies. The reduced value is found already one hour after the tracer injection, and the value is almost unchanged after 2 and 4 h. Furthermore, the kidney/background ratio was already reduced at one month to a level that was maintained after 3 and 6 months.

The objective of the present study was to examine the influence of procedural variables associated with ^123^I-MIBG imaging when a standardized technique was used to determine the kidney/background ratio. However, in the absence of guidelines for renal ^123^I-MIBG imaging, there has been limited standardization of the acquisition and quantitative analysis techniques, and published studies have involved small numbers of subjects and different reference standards. Therefore, in the attempt to evaluate the ^123^I-MIBG method’s ability to assess renal denervation, we used an acquisition protocol adapted from cardiac Metaiodobenzylguanidine (MIBG) studies using early (15 min post-injection) planar and SPECT/CT and late (1, 2, and 4 h post-injection) planar imaging. While cardiac MIBG images at 15 min provide an indication of net extraction efficiency by sympathetic neurons (albeit with contamination by blood pool activity), this study revealed that renal counts at this time point predominantly reflected glomerular filtration and urinary excretion of MIBG. This is evident in the washout data in Figure 4 and Table 2, where the high calculated rate between 15 min and 1 h (as much as 6 times greater than the rate between 1 and 2 h) undoubtedly reflects urinary excretion during the early time interval. Retrospectively, it is unfortunate that SPECT/CT was only performed at the early time point, in that the previously noted problem with urinary contamination limits the reliability of global or regional quantitation from these images.

The observed 30% reduction in relative 4 h ^123^I-MIBG uptake in the recipient kidneys one month after transplantation in our study is in line with a previously published case report with measurement two weeks after auto-transplantation of a kidney [25]. Renal sympathetic neurons may however represent only a negligible portion of this ^123^I-MIBG uptake. Supporting this, is one study of liver transplant patients in which norepinephrine concentration in liver biopsies one month post-transplantation was only 1% of that in healthy normal livers [31]. In line with this and the interpretation by Dobrowlski and colleagues [24], scintigraphic values from the kidneys mainly comprise simultaneous uptake and storage of ^123^I-MIBG as well as an ongoing excretion of ^123^I-MIBG. The notable very low washout rate at 3 and 6 months between 2 and 4 h is due to some patients who had a negative washout in that time interval. It is yet to be established which time interval is the most optimal to measure washout rate. The assumed activity during the early phase following ^123^I-MIBG injection will represent a mixture of content in the pre-urine and washout from the renal tissue [16,24]. The washout rate of ^123^I-MIBG in kidneys might be difficult to untangle from filtration and, therefore, may be less relevant to estimating renal sympathetic activity.

### 4.1. Strengths and Limitations

In the present study we used the same kidney within the live kidney donor before transplantation as the control value, whereas in the only other study of renal ^123^I-MIBG scintigraphy in kidney transplant recipients, hypertensive non-transplant patients were used as controls [24]. Furthermore, by using attenuation correction we sought to eliminate the effect of the kidney being moved not only from one person to another, but also with an altered position within the body. The transplantation model implies a state of complete renal denervation, whereas in patients who have a catheter-based renal denervation, expected to be only partial, no significant change in renal ^123^I-MIBG scintigraphy uptake or washout have been demonstrated at 6 weeks after denervation [26].

It may be a limitation of our study that participants were kept on medications that possibly could influence the interpretation of the ^123^I-MIBG scintigraphy. In addition, an inherit challenge with imaging acquisition is the scatter from uptake by the liver with the right-sided native kidneys at baseline, and from the bladder with the transplanted kidneys. We assume that only denervation contributes to alterations in renal ^123^I-MIBG scintigraphy, but in the post-transplant phase patients are treated with immune suppressant medication that potentially may influence renal ^123^I-MIBG kinetics and sympathetic nerve function.

We acknowledge the absence of standardized methods for quantifying MIBG uptake in each of the three main components of the kidneys, namely, vascular, parenchymal (in which neuronal uptake is a part), and urinary uptake. Therefore, in the absence of independent measures of renal blood flow and urinary excretory function our results are limited to reflect MIBG uptake in these compartments as a whole and not specifically in sympathetic neurons.

Retrospectively, it is a limitation that we did not use SPECT/CT at all time points for determination of attenuation-corrected regional renal activity, which would have generated more accurate results compared to the use of the relative kidney uptake measurement, adapted from the planar heart/mediastinum ratio.

It is of course a limitation that this is a small study. Nevertheless, our results are consistently showing the same trend with statistical significance.

### 4.2. Future

In terms of the neuroanatomy of the nerves, Wallerian degeneration, neuronal regrowth, and the results in this study, it would be interesting to follow live-donor transplanted kidneys for a longer period in order to monitor changes in ^123^I-MIBG uptake. A recent study of patients who have had a heart transplantation shows that using three different catecholaminergic tracers simultaneously can evaluate the separate functions of the sympathetic nerve terminal such as; (a) neuronal transport, (b) vesicular storage; and (c) metabolic degradation [32]. It would be of interest to use this combined tracer technique to evaluate corresponding functions of denervated kidneys.

## 5. Conclusions

To our knowledge, the present study is the first in the setting with ^123^I-MIBG uptake measurements of the same kidney before and after complete denervation due to live kidney transplantation. Live kidney transplantation was associated with a 30–40% reduction in the relative 4 h ^123^I-MIBG uptake of the kidney. The renal ^123^I-MIBG scintigraphy method has to be further refined to fully establish the interpretation and its implications. A marker of renal nervous activity would be of great value in both chronic kidney disease, renal denervation, and renal transplantation, both in recipients and donors.

## Figures and Tables

**Table 1:** Baseline clinical characteristics.

	Donors	Recipients
Age, years (SD)	52 (13)	47 (16)
Gender, female, *n* (%)	4 (45)	5 (36)
^51^Cr-EDTA clearance, ml/min (SD)	106 (28)	*
Office blood pressure, systolic/diastolic, mmHg (SD)	135 (15)/83 (10)	139 (25)/82 (15)
Diabetes, *n* (%)	0 (0)	3 (27)
Hypertension, *n* (%)	0 (0)	3 (27)

^51^Cr-EDTA: Chromium-51-Ethylenediaminetetraacetic acid; SD: Standard deviation. Values are shown as mean (SD) or numbers (%). * Recipients did not have a ^51^Cr-EDTA plasma clearance performed routinely prior to transplantation as they, per definition, had an eGFR of ≤15 mL/min*1.73m^2^ and/or required chronic dialysis. The reasons to perform the kidney transplantation and medicine status figures are in the Supplementary Material (Table S1).

**Table 2:** Relative ^123^I-MIBG uptake and washout rate in the donor kidney over time.

	Baseline *n* = 11 (SD)	1 Month *n* = 11 (SD)	3 Months *n* = 10 (SD)	6 Months *n* = 11 (SD)	Change between Baseline and 6 Months Mean (95% CI)	*p* Value
**Relative Ratio**						
15 min Kidney/Background	6.8 (1.8)	5.0 (1.4)	5.5 (1.4)	4.7 (1.5)	−2.3 (−3.4 to −1.2)	0.0010 *
1 h Kidney/Background	4.8 (0.9)	3.3 (1.0)	3.6 (0.8)	3.1 (0.6)	−1.8 (−2.7 to −0.9)	0.0012 *
2 h Kidney/Background	4.5 (0.6)	3.0 (0.5)	3.1 (0.5)	2.7 (0.5)	−1.9 (−2.6 to −1.2)	0.0002 *
4 h Kidney/Background	4.0 (0.4)	2.7 (0.4)	2.7 (0.5)	2.5 (0.4)	−1.5 (−2.0 to −1.0)	<0.0001 *
**Washout Rate**						
15 min to 1 h (%/hour)	50.1 (42.7)	42.6 (25.5)	50.9 (26.2)	31.3 (33.5)	−16.9 (−38.7 to 4.8)	0.1109
15 min to 2 h (%/hour)	26.8 (21.8)	23.4 (14.0)	31.0 (10.4)	27.5 (12.8)	−0.7 (−14.8 to 13.4)	0.9067
15 min to 4 h (%/hour)	16.3 (9.4)	15.2 (4.2)	15.5 (5.7)	13.7 (7.2)	−3.3 (−9.5 to 2.7)	0.2436
1 to 2 h (%/hour)	9.6 (9.8)	7.3 (28.6)	16.8 (14.8)	22.7 (20.9)	10.1 (−10.0 to 30.3)	0.2731
2 to 4 h (%/hour)	9.4 (5.0)	7.8 (11.9)	1.5 (9.6)	1.8 (6.0)	−8.3 (−15.1 to −1.4)	0.024 *
**Washout**						
Washout 15 min to 4 h (%)	43.3 (14.7)	42.8 (9.1)	42.7 (11.8)	38.1 (18.0)	−6.7 (−19.8 to 6.4)	0.2800

Results are given as mean and standard deviation (SD). Relative ^123^I-MIBG uptake: Comparing the relative ^123^I-MIBG uptake before and 6 months following transplantation there is a significant reduction in estimation of renal sympathetic activity on all measurement points. Washout rate: Within each of the five individual time intervals there was no statistical difference between baseline values to 6 months following transplantation. K/B; kidney/background. CI: Confidence Interval. * Statistical significant reduction compared to the baseline value.

## Supplementary Materials

The following are available online at https://www.mdpi.com/2075-4418/10/10/802/s1, Table S1. Detailed information of the 11 sets of donor/recipients with concern to kidney function, medication, and co-morbidity.

## Appendix A

### Attenuation Correction

Since arms are not covering the patient’s body in the anterior–posterior direction, it was assumed that the CT with arms above the head could be used for attenuation correction with arms along the patient’s body. The CT-image was integrated in the anterior–posterior direction (z-direction) resulting in a CT water equivalent thickness T (including the patient table) given in cm by

(A1) $$T_{\mathrm{water}}=\int^{\;}\left(1+\mathrm{kCT}\right)\mathrm{dz}=\int_{0}^{T}\frac{\mu -\mu_{\mathrm{air}}}{\mu_{\mathrm{water}}-\mu_{\mathrm{air}}}\mathrm{dz}$$

where kCT indicates the CT number divided by 1000. CT numbers (in Hounsfield Units = HU) were converted into attenuation coefficients for 159 keV photons by bilinear interpolation [33] with a transition point at 100 HU. Three calibration points were taken for air, water (1.00 g/cm^3^), and Teflon (2.20 g/cm^3^). The corresponding CT numbers were set at −1000, 0, and 896 HU, respectively. The Teflon value is slightly keV dependent [34]. The corresponding narrow beam attenuation coefficients for 159 keV photons were 0.000, 0.1477, and 0.2827 cm^-1^, respectively. The water and Teflon values were obtained from a double logarithmic fit and interpolation between 150 and 200 keV on the data from the National Institute of Standards and Technology [35]. The geometric mean, also called the conjugated image, of the anterior A and (left–right mirrored) posterior image *p* was calculated with G = √AP. The attenuation factor AC was calculated for every pixel with:

(A2) $$\mathrm{AC}=e^{-\frac{1}{2}\int^{\;}\mu \mathrm{dz}}$$

This is derived for a point source position at an arbitrary position along the z-direction. The attenuation corrected geometric image G_AC_ was created by multiplying AC with the geometrical mean, G_AC_ = G*A. All calculations were performed in Matlab 2014b (Mathworks, Natick, MA, USA).
