# Supplementary material for: Renal 123I-MIBG Uptake before and after Live-Donor Kidney Transplantation
Source: Diagnostics (Basel). 2020 Oct 9;10(10):802. doi: 10.3390/diagnostics10100802 (PMC7601882; doi:10.3390/diagnostics10100802)
Supplement: Supplementary file 1 [file diagnostics-10-00802-s001.pdf]

diagnostics

MDPI

Table S1. Detailed information of the 11 sets of donor/recipients with concern to kidney function, medication, and co-morbidity.

	**Gender**<br>M: Male<br>F: Female	**Age**<br>(years)	**Renography**<br>(Right/left, %)	**^{51}Cr-EDTA Clearance**		**Co-Morbidity**<br>Diabetes (DM).<br>Hypertension (HTN).<br>Ischemic Heart Disease (IHD).<br>Cerebral Vascular Insult (CVI).	**Medication that Potentially Could Influence ^{123}I-MIBG Uptake**				**RECIPIENTS**
				(mL/min)	(ml/min*1, 73m^2)		**Before**	**1 month after**	**3 months after**	**6 months after**	**1) Cause of Renal Failure**<br>**2) Pre-Transplantation Dialysis**<br>**3) Previous Transplantation (Year)**<br>**4) Rejection of Current Donor Kidney**
**Donor I**	M	74	37/63	85	70	No DM. No HTN.<br>No IHD. No CVI.	-	Amlodipine 5 mg<br>Furosemide 20 mg	Amlodipine 5 mg<br>Furosemide 20 mg	Amlodipine 5 mg<br>Furosemide 20 mg	1) Hypertension<br>2) No<br>3) No<br>4) No
**Recipient I**	F	70	-	-	-	No DM. No IHD. No CVI<br>+HTN	-	Metoprolol 200 mg	Metoprolol 200 mg	Metoprolol 200 mg	
**Donor II**	F	44	47/53	98	93	No DM. No HTN.<br>No IHD. No CVI.	-	-	-	-	1) FSGS (mutation NPHS2-genet) age 6 y.<br>2) Hemodialysis, 6 months<br>3) No (native kidneys nephrectomized)<br>4) Rejection (grade IIA, 3 days post Tx)
**Recipient II**	M	21	-	-	-	No DM. No HTN.<br>No IHD. No CVI.	-	Furosemide 80 mg	Furosemide 80 mg	Furosemide 40 mg	
**Donor III**	F	44	42/58	92	92	No DM. No HTN.<br>No IHD. No CVI.	-	-	-	-	1) Diabetic nephropathy<br>2) No<br>3) No<br>4) Rejection (grade 1B, 11 months post Tx)
**Recipient III**	M	50	-	-	-	No HTN. No IHD. No CVI<br>+DM	-	-	-	Furosemide 40 mg	
**Donor IV**	M	37	54/46	163	111	No DM. No HTN.<br>No IHD. No CVI.	Morphine 20 mg<br>Paroxetine 20 mg<br>Nortriptyline 25 mg	Morphine 60 mg<br>Paroxetine 20 mg<br>Nortriptyline 25 mg	Morphine 60 mg<br>Paroxetine 20 mg<br>Nortriptyline 25 mg	Morphine 60 mg<br>Paroxetine 20 mg<br>Nortriptyline 25 mg	1) Congenital structural abnormality<br>2) Hemodialysis (2006–2014)<br>3) One (1999–2006, chronic rejection)<br>4) No
**Recipient IV**	M	46	-	-	-	No DM. No HTN.<br>No IHD. No CVI.	-	Furosemide 80 mg	Furosemide 80 mg	Furosemide 80 mg	
**Donor V**	M	68	43/57	79	75	No DM. No HTN.	-	-	-	Losartan 50 mg	1) Polycystic kidney disease

						No IHD. No CVI.					2) No<br>3) No<br>4) Rejection (grade IIA, 5 days post Tx)
**Recipient V**	F	46	-	-	-	No DM. No IHD. No CVI.<br>+HTN	-	Felodipine 10 mg	Felodipine 10 mg	Felodipine 5 mg<br>Metoprolol 100 mg	
**Donor VI**	M	40	45/55	151	110	No DM. No HTN.<br>No IHD. No CVI.	-	-	-	-	1) Diabetic nephropathy (diagnosed 1979)<br>2) Hemodialysis (2010-2014)<br>3) No<br>4) Rejection (grade IIA; 1,5 month post Tx)
**Recipient VI**	F	43	-	-	-	No DM. No HTN.<br>No IHD. No CVI.	-	-	-	-	
**Donor VII**	F	68	49/51	79	79	No DM. No HTN.<br>No IHD. No CVI.	-	-	-	-	1) Nephrotic syndrome, minimal change<br>2) Hemodialysis (2010–2014)<br>3) No<br>4) No
**Recipient VII**	M	69	-	-	-	No IHD. No CVI.<br>+ DM. + HTN.	-	Losartan 100 mg<br>Felodipine 10 mg<br>Furosemide 100 mg	Losartan 100 mg<br>Felodipine 10 mg<br>Furosemide 100 mg	Losartan 100 mg<br>Felodipine 10 mg<br>Furosemide 100 mg	
**Donor VIII**	M	55	55/45	108	85	No DM. No HTN.<br>No IHD. No CVI.	-	-	-	-	1) FSGS (1991)<br>2) No<br>3) No<br>4) No
**Recipient VIII**	F	51	-	-	-	No DM. No HTN.<br>No IHD. No CVI.	-	-	Metoprolol 150 mg	Metoprolol 150 mg	
**Donor IX**	M	42	60/40	104	86	No DM. No HTN.<br>No IHD. No CVI.	-	-	-	-	1) Diabetic nephropathy<br>2) Peritoneal dialysis (2014-2015)<br>3) No<br>4) Rejection (grade IA;3 days post Tx)
**Recipient IX**	M	41	-	-	-	No HTN. No IHD. No CVI.<br>+ DM	-	-	-	Metoprolol 100 mg	
**Donor X**	M	51	47/53	97	121	No DM. No HTN.<br>No IHD. No CVI.	-	-	-	-	1) Traumatic based nephrectomy (2014)<br>2) Hemodialysis (2014–2015)<br>3) No<br>4) No
**Recipient X**	F	19	-	-	-	No DM. No HTN.<br>No IHD. No CVI.	-	Furosemide 20 mg	-	-	
**Donor XI**	F	51	47/53	86	94	No DM. No HTN.	-	-	-	-	1) FSGS (2000)

						No IHD. No CVI.					2) No
**Recipient XI**	M	56	-	-	-	No DM. No HTN.<br>No IHD. No CVI.	-	-	-	-	3) No<br>4) No

[51]Cr-EDTA: Chromium-51-Ethylenediaminetetraacetic acid; CVI: Cerebral Vascular Insult; DM: Diabetes Mellitus; FSGS: Focal Segmental Glomerulosclerosis; HTN: Hypertension; IHD: Ischemic Heart Disease: Tx: Transplantation
